# The effect of laboratory-verified smoking on SARS-CoV-2 infection: results from the Troina sero-epidemiological survey

**DOI:** 10.1007/s11739-022-02975-1

**Published:** 2022-04-14

**Authors:** Venera Tomaselli, Pietro Ferrara, Giulio G. Cantone, Alba C. Romeo, Sonja Rust, Daniela Saitta, Filippo Caraci, Corrado Romano, Murugesan Thangaraju, Pietro Zuccarello, Jed Rose, Margherita Ferrante, Jonathan Belsey, Fabio Cibella, Grazia Caci, Raffaele Ferri, Riccardo Polosa

**Affiliations:** 1 Department of Political and Social Sciences, University of Catania, Catania, Italy; 2 Center of Excellence for the Acceleration of Harm Reduction (CoEHAR), University di Catania, Catania, Italy; 3 Department of Public Health, Experimental and Forensic Medicine, University of Pavia, Pavia, Italy; 4 Center for Public Health Research, University of Milan-Bicocca, Monza, Italy; 5 Department of Physics and Astronomy “Ettore Majorana”, University of Catania, Catania, Italy; 6 Oasi Research Institute-IRCCS, Troina, Italy; 7 ECLAT Srl, Spin-off of the University of Catania, Catania, Italy; 8 Department of Drug and Health Sciences, University of Catania, Catania, Italy; 9 Bioanalytical Laboratory, Center for Smoking Cessation, Duke University Medical Center, Durham, USA; 10 Department of Psychiatry and Behavioral Sciences, Duke University Medical Center, Durham, USA; 11 Department of Medical, Surgical Sciences and Advanced Technologies “G.F. Ingrassia”, University of Catania, Catania, Italy; 12 JB Medical Ltd, Sudbury, UK; 13 Institute of Biomedicine and Molecular Immunology, National Research Council of Italy, Palermo, Italy; 14 Unit of Infectious Diseases, Department of Clinical and Experimental Medicine, University of Messina, Messina, Italy; 15 Department of Clinical and Experimental Medicine, University of Catania, Catania, Italy; 16 Institute of Internal Medicine, AOU “Policlinico-V. Emanuele”, Via S. Sofia, 78, Catania, Italy

**Keywords:** Antibody persistence, Cotinine, COVID-19, SARS-CoV-2-specific immunoglobulin, Sero-prevalence, Smoking

## Abstract

Previous research yielded conflicting results on the association between cigarette smoking and risk of SARS-CoV-2 infection. Since the prevalence of smoking is high globally, the study of its impact on COVID-19 pandemic may have considerable implications for public health. This study is the first to investigate the association between the SARS-CoV-2 antibody sero-positivity and biochemically verified smoking status, to refine current estimates on this association. SARS-CoV-2-specific IgG and serum cotinine levels (a well-known marker of tobacco exposure) were assessed in a large sero-epidemiological survey conducted in the town of Troina (Sicily, Italy). A propensity score matching was carried out to reduce the effect of possible factors on SARS-CoV-2 infection risk among study participants. Of the 1785 subjects included in our study, one-third was classified as current smokers, based on serum cotinine levels. The overall proportion of subjects with positive serology for SARS-CoV-2 IgG was 5.4%. The prevalence of SARS-CoV-2 antibody positivity and previous COVID-19 diagnosis were reduced in smokers. This reduced prevalence persisted after adjusting for possible confounders (such as sex, age, previous infection, chronic conditions, and risk group) at regression analyses, and the point estimates based on the PS-matched models resulted consistent with those for the unmatched population. This study found a lower proportion of positive SARS-CoV-2 serology among current smokers, using direct laboratory measures of tobacco exposure and thus avoiding possible bias associated with self-reported smoking status. Results may also serve as a reference for future clinical research on potential pharmaceutical role of nicotine or nicotinic-cholinergic agonists against COVID-19.

**Supplementary Information:**

The online version contains supplementary material available at 10.1007/s11739-022-02975-1.

## Introduction

The severe acute respiratory syndrome—coronavirus 2 (SARS-CoV-2) pandemic represents a major public health challenge, having resulted in almost 395 million confirmed infections and 5.7 million deaths reported to the World Health Organization in the last 2 years, with relative societal and healthcare system demands [1–3].

Since the spread of the first coronavirus disease 2019 (COVID-19) cases—the disease associated with SARS-CoV-2 infection—in late 2019, the pandemic has spotlighted the essential need for research efforts towards understanding the clinical features of the disease, as well as to develop treatments and vaccines to tackle this health emergency [4–6]. As the global burden of COVID-19 continues to rise, observational evidence is focusing on the study of possible predictors and risk factors for SARS-CoV-2 infection and COVID-19 outcomes [5, 7–12].

Approximately 1.3 billion people worldwide use tobacco, of which more than 7 million die prematurely every year [13]. Smoking is the main cause of lung cancer, chronic obstructive pulmonary disease, and cardiovascular disease [14–16].

Smoking is also a significant risk factor for both viral and bacterial infections of the respiratory system [17, 18], and the association between smoking and COVID-19 has caught the attention of the research community from the very first epidemic weeks [19, 20].

Surprisingly current research on the relationship between smoking and SARS-CoV-2/COVID-19 has yielded conflicting findings [19–21]. For instance, a positive association between smoking and risk of COVID-19-related severe outcomes, hospitalization and death was found in some primary and secondary studies [22–25]. In contrast, other studies have identified lower proportions of active smokers among patients diagnosed with SARS-CoV-2 [26–29], and a significant lower prevalence of smoking among hospitalized COVID-19 patients than that expected on the basis of population smoking prevalence [30, 31]. On both hypotheses, the literature so far available is affected by several design issues, which make studies hard to compare [30–32]. In particular, many reports derived data on smoking from case-series, where the chance of inaccurate recording of smoking status/history, or other recall/reporting bias cannot be excluded, in consideration of the challenging circumstances in which data were collected [19, 33]. Recently, Farsalinos et al. looked at a possible systematic ascertainment bias in determining the overall smoking-related risk across the research that reported a higher risk for severe COVID-19 in hospitalized smokers [20]. These authors suggested that, if smokers might be less likely to develop the infection or severe disease [27–29], a higher risk for adverse outcome among hospitalized smokers is not applicable to all smokers but only to the small proportion of smokers who end up being hospitalized due to COVID-19 [20].

Additional concerns have been raised about the possible sources of residual confounding. Some studies relied on self-reported smoking habits and most failed to correct for relevant confounders which could result in different risk exposure while others combined current smokers and former smokers into one category, preventing from examining the interaction of SARS-CoV-2 with active smoking and its effect on risk of infection and COVID-19 outcomes [11, 22, 24, 34]. As a whole, all these limitations reflect problems with poor reporting of the smoking status as well as lack of studies specifically designed to examine the association between smoking and COVID-19. All these factors could affect the conclusions derived from the existing body of evidence [19].

To address potential bias in estimation of smoking prevalence by self-reporting, we designed a sero-prevalence study—an attempt to measure the true infection rates in selected populations [26, 35]—to investigate the association between the SARS-CoV-2 antibody sero-positivity and biochemically verified smoking. Serum cotinine measurements were used, which is a well-known marker of tobacco exposure since cotinine is the predominant metabolite of nicotine, and cotinine assays are generally used to discriminate active smokers from non-smokers [36].

## Materials and methods

### Study design, setting, and population

The Troina project defines a cohort study conducted between July and September 2020. The complete study protocol has been previously published [33]. Surveillance of antibody sero-positivity and biochemically verified smoking status (i.e., cotinine) was conducted in serum samples of the general population and healthcare workers (HCW) from the town of Troina (Province of Enna, Sicily, Italy). Troina has a population of over 9000 inhabitants and in March 2020 censused a high incidence of SARS-CoV-2 infections during the early weeks of the first wave of the pandemic, prompting local authorities to declare the town a ‘red zone’ and to enforce lockdown rules on March 29, although no relevant difference in hospitalization and mortality rates was registered compared to regional data [33, 37].

The study population consisted of two groups: (i) a population-based, gender and age-stratified cohort randomly selected from the population registry of town residents; (ii) a convenience sample of hospital care workers (HCW) of Troina’s main health facility (IRCCS Oasi Maria Santissima, Troina, Enna, Italy), who were likely to have been in close contact with COVID-19 patients and, therefore, at high risk of infection. Any individual, irrespective of age, who lived in the town of Troina or worked in the selected institution was eligible for inclusion in the study. Subjects who refused to provide informed consent or had contraindication to venepuncture were excluded. Suspected or confirmed active/acute, recent, or prior SARS-CoV-2 infection was not considered as an exclusion criterion for this investigation. For individuals receiving medical care for COVID-19 during the study period, a proxy respondent (i.e., family member) was contacted to gather questionnaire responses.

The cohort assembly and sample size determination followed the WHO protocol for sero-epidemiological investigation for SARS-CoV-2 infection [38], and has been described in detail previously [33]. Briefly, to aim for a representative sample of the population by gender and age groups, a targeted sample size for this study was specified for each category. We, therefore, calculated several sample sizes depending on the margin of error equal or minor than 3% for estimate proportions of sampled population. Consequently, a sample of subjects was sought in the entire population. The attrition rate was fixed at 10%. In the second group, all workers from the selected institution were invited to participate in the study. Participation was voluntary; enrolees were not offered any incentive for and were informed about their right to drop out of the study at any time for any reason or no reason at all, without penalty. Each participant received complete information about the nature and protocol of the research, and informed that all information gathered would be anonymous and confidentiality would be maintained by omitting any personal identifying information. All participants provided informed consent at the enrolment; in case of participants aged less than 18 years, consent was obtained from their parents.

The Research Ethics Committee of the IRCCS Oasi Maria Santissima (Troina, Enna, Italy) approved the research protocol, survey instruments, and informed consent form (approval n. 11/2020).

### Study endpoints and data definitions

This study was conducted to: (i) measure the serum concentration of anti-SARS-CoV-2 immunoglobulin classes G (IgG) in the study sample, to quantify the prevalence of subjects with altered immunologic profile due to SARS-CoV-2 infection since the beginning of the pandemic; (ii) evaluate the level of serum cotinine, to quantify the proportion of biochemically verified current, former and never smokers; (iii) analyse the relationship between active smoking (current vs. non-smokers) and SARS-CoV-2 infection. Secondary outcomes included the assessment of: (i) the proportion of participants with of COVID-19-like symptoms; (ii) the prevalence of SARS-CoV-2 confirmed diagnosis in the study sample; (iii) the proportion of subjects hospitalized due to COVID-19; (iv) recording relevant clinical confounders known to be associated with SARS-CoV-2 infection risk and COVID-19 outcomes (including sex, age, occupational exposure to SARS-CoV-2). Smoking was defined as any use of tobacco cigarettes, cigars and/or rolls. Current smokers were defined as those who reported active smoking and with serum cotinine levels ≥ 20 ng/mL; former smokers were those who reported that they smoked in the past, but not at the time of the survey and had serum cotinine levels < 20 ng/mL; never smokers those who reported that they never smoked and had serum cotinine levels < 20 ng/mL [39].

### Study procedures

Details about participants’ interviews and blood specimen handling (collection, transport, aliquoting, biobanking, storage, and assays) have been previously described [33]. In brief, a one-site testing-point was set up with trained personnel, where participants were interviewed regarding demographics and professional characteristics (sex, age, working status), health status (presence of comorbidities, use of medications), history of smoking and smoking habits, history of symptoms compatible with COVID-19 (i.e., fever, severe tiredness, sore throat, cough, shortness of breath, headache, anosmia, ageusia, nausea, vomiting, diarrhoea, or any other COVID-19-like symptom), previous diagnosis with SARS-CoV-2 and/or need of medical contacts or hospitalization due to COVID-19. Subsequently, collection of a blood sample (10 mL) by venepuncture was performed on each participant upon at the same testing-point. For each specimen, the time of collection, the conditions for transportation and the time of arrival at the study laboratory were recorded. Specimens reached the laboratory as soon as possible after collection, where serum was separated from whole blood and stored at − 80 °C until use.

Serum samples were screened for anti-SARS-CoV-2 human antibodies using the EUROIMMUN Anti-SARS-CoV-2 Assay, an enzyme-linked immunosorbent assay (ELISA) that provides semi-quantitative in vitro determination of neutralizing IgG that bind the SARS-CoV-2 spike (S) protein receptor binding domain (RBD)—the most critical target for SARS-CoV-2-specific immunoglobulin within the S1 sub-unit [40]. According to the manufacturer’s recommendations, positivity was intended a ratio equal to or greater than 1.1 [41]. The EUROIMMUN Anti-SARS-CoV-2 ELISA test presented sensitivity of 100% (95% CI 91.6–100) and specificity of 97.7% (95% CI 91.9–99.6) four days after COVID-19 diagnosis by real-time polymerase chain reaction (PCR) [42].

For the evaluation of cotinine, pre-conditioned samples were injected into HP-5 Capillary GC Column (0.32 mm ID, 25 m length and 0.52 μm film thickness; bonded 5% phenyl and 95% dimethylpolysiloxane) of GC-NPD. Complete procedures can be found in the study protocol [33]. Serum levels of cotinine were reported in ng/mL. The limit of quantification of cotinine with GC-NPD is 20 ng/mL. The 20 ng/mL value is a very reasonable cut-off for serum cotinine to distinguish smokers from non-smokers [39].

### Statistical analysis

Continuous variables were expressed as mean (standard deviation, SD); categorical variables were described as number and percentage. Differences between continuous variables were evaluated though Student *t* test or Mann–Whitney *U* test according to their distribution; the chi-squared (*χ*^2^) test and Fisher exact test to assess differences among categorical variables. Comparisons were examined among subjects who tested negative and positive to SARS-CoV-2 antibodies. Considering the imbalance of the covariates across smokers’ groups, we performed a propensity score matching (PSM), accounting for those characteristics that were likely to have had an effect on the risk of SARS-CoV-2 infection. The following variables were selected: age, sex, presence of comorbidities (at least, one important chronic condition), cohort group (as proxy of exposure risk to SARS-CoV-2). The propensity score for smoking was calculated using the logistic regression model. According to optimal PSM match ratio and calliper widths for the estimation of differences in means and proportions in observational studies [43, 44], we matched the respondents on a 1:1 ratio, using the nearest neighbouring method with a calliper matching of 0.2. Complete PSM methodology is presented in the Supplementary material.

Subsequently, we fitted multinomial logistic regression analyses to model the association between the outcomes of interest and active smoking, adjusting for possible covariates (age, sex, presence of at least one comorbidity, exposure group, and presence of COVID-19-like symptoms) both in the unmatched and matched study cohorts. The following models were constructed: likelihood of testing positive at SARS-CoV-2 serology (Model 1); likelihood of having received the diagnosis of SARS-CoV-2 infection (Model 2); likelihood of being hospitalized due to COVID-19 (Model 3). The latter, due to the small number of observations which did not allow at making inference on the asymptotic results as in logistic regressions, was built as an exact logistic regression for small samples, in which the log odds of the outcome is modelled as a combination of the predictor variables [45]. Results were reported as adjusted odds ratios (aOR) with 95% confidence intervals (CI). Based on similar approaches in the literature [46], a multivariate Poisson regression analysis with log-link was conducted to model the incidence of COVID-19-like symptoms, as a function of baseline participant characteristics and smoking status. This enabled the relative risks (as incidence rate ratios, RR) associated with a set of covariates to be estimated, including age, sex, and presence of comorbidities. All *p* values were two-sided and < 0.05 assumed as statistically significant. Statistical analyses were conducted with statistical software STATA version 17 (StataCorp. 2021, College Station, TX, USA) and *R* version 3.6.2 (*R* Project for Statistical Computing, Vienna, Austria).

## Results

### Study population

**Fig. 1:**
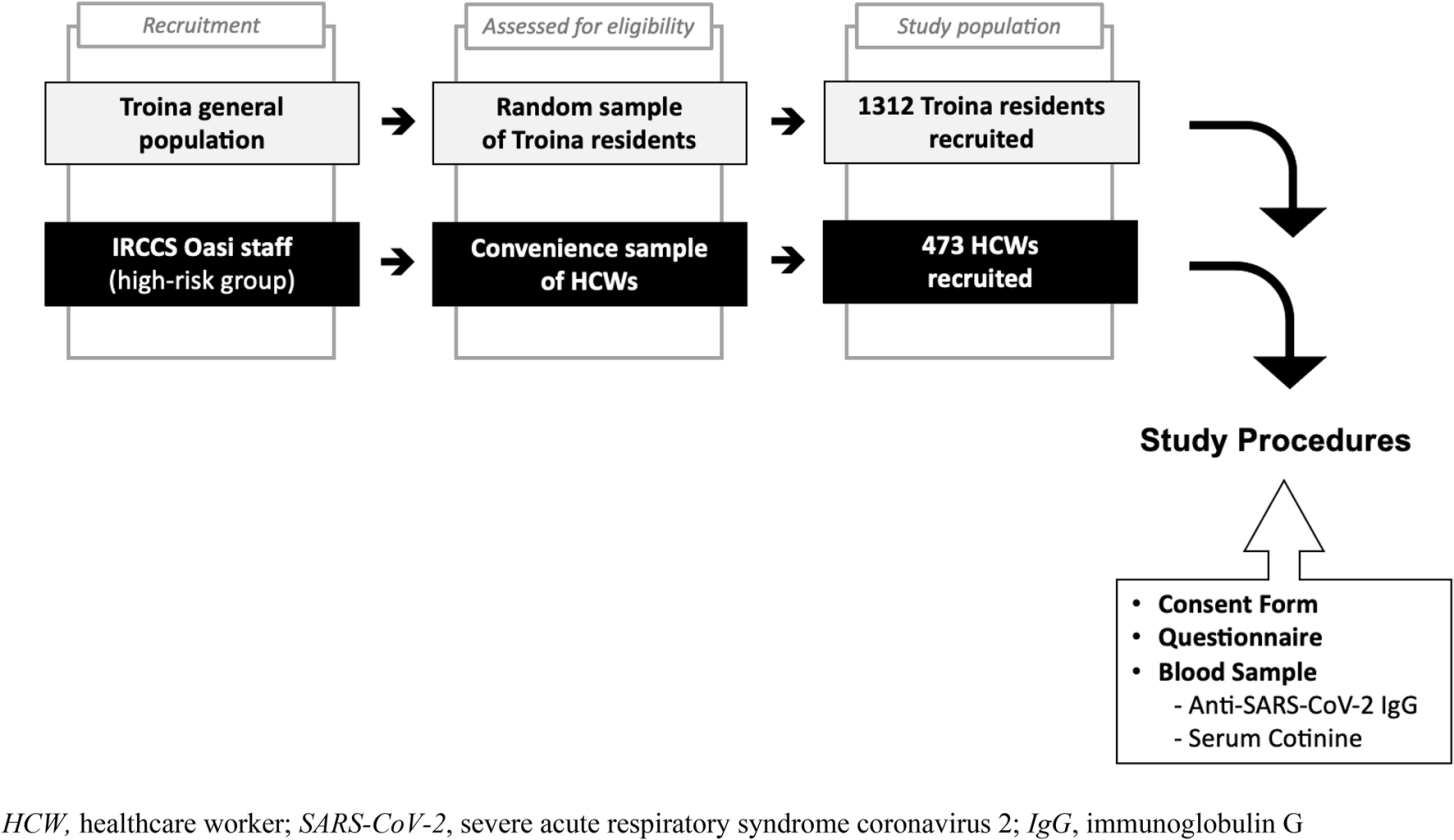
The Troina Study flow diagram. *HCW *healthcare worker, *SARS-CoV-2* severe acute respiratory syndrome coronavirus 2, *IgG* immunoglobulin G

After validating the self-reported smoking status with the serum cotinine threshold to distinguish smokers from non-smokers, a total of 1785 cotinine-verified subjects entered the study: 1312 (73.5%) subjects were sampled from the town of Troina; while 473 (26.5%) constituted the HCW population enrolled in the Troina’s main health care facility. The flowchart of the cohort creation is presented as Fig. 1. Specifically, the majority of participants was female (61.4%), with a mean age of 50 years, and 56.1% had at least one chronic disease. The baseline characteristics of the study population are presented in Table 1, stratified by enrolment group.

**Table 1:** Characteristics of the study population by enrolment group

Characteristic	Total *N* (%)	General population *N* (%)	Healthcare workers *N* (%)
	1785	1312 (73.5)^a^	473 (26.5)^a^
Sex			
Female	1096 (61.4)	750 (57.2)	346 (73.2)
Male	689 (38.6)	562 (42.8)	127 (26.8)
Age (in years)^b^	50.0 ± 19.7	50.4 ± 22.2	48.9 ± 9.7
Working status			–
Occupied	916 (51.3)	444 (33.8)	
Student	197 (11.0)	197 (15.0)	
Unoccupied	306 (17.1)	306 (23.3)	
Retired	366 (20.5)	365 (27.8)	
Smoking history			
Current smoker	543 (30.4)	369 (28.1)	174 (36.8)
Former and never smoker	1242 (69.6)	943 (71.9)	299 (63.2)
Smoking time (in years)^b^	20.4 ± 14.5	19.8 ± 15.2	21.8 ± 12.8
Comorbidities^c^			
At least one	1000 (56.1)	772 (58.8)	228 (48.3)
Heart disease	152 (15.2)	141 (18.3)	11 (4.8)
Vascular pathologies	64 (6.5)	48 (6.2)	16 (7.0)
Cerebrovascular disease	5 (0.5)	4 (0.5)	1 (0.4)
Diabetes mellitus	92 (9.2)	80 (10.4)	12 (5.3)
Hypertension	405 (40.5)	331 (42.9)	74 (32.5)
Respiratory pathologies	20 (2.0)	15 (1.9)	5 (2.2)
Bronchial asthma	25 (2.5)	20 (2.6)	5 (2.2)
Chronic kidney disease	8 (0.8)	4 (0.5)	4 (1.8)
Cancer	20 (2.0)	10 (1.3)	10 (4.4)
Autoimmune disease	49 (4.9)	16 (2.1)	33 (14.5)
Mental disorder	5 (5.0)	5 (0.6)	0 (0.0)
Organ transplant history	2 (2.0)	2 (0.3)	0 (0.0)
Others	681 (68.1)	536 (69.4)	145 (63.6)

^a^Row percentage

^b^Summarized by mean and standard deviation (SD)

^c^Percentage was calculated on subjects with at least one chronic condition

### SARS-CoV-2 infection and smoking

The overall proportion of subjects with positive serology for SARS-CoV-2-specific IgG was 5.4%. Respondents’ characteristics according to the positivity at antibody testing are listed in Table 2. No significant sex or age differences in antibody response were observed. As expected, HCWs showed a higher prevalence of IgG positivity than general population (72.9 vs. 27.1%, *p* value < 0.001). A difference in positive serology rate among those who experienced at least one COVID-19-like symptom after March 1, 2020 and those who did not was also present (65.6 *vs.* 11.4%; *p* value < 0.001); the found increased rate was confirmed for each of the reported symptoms (*p* value < 0.001 for all).

**Table 2:** Characteristics of the study population stratified by the presence of antibodies for SARS-CoV-2 infection

Characteristic	Total *N* (%)	SARS-CoV-2 IgG positive *N* (%)	SARS-CoV-2 IgG negative *N* (%)	Comparison (*p* value)
*N*	1785	96 (5.4)^a^	1689 (94.6)^a^	
Sex				0.19
Female	1096 (61.4)	65 (67.7)	1031 (61.0)	
Male	689 (38.6)	31 (32.3)	658 (39.0)	
Age (in years)^b^	50.0 ± 19.7	48.6 ± 15.7	50.0 ± 19.9	0.49
Working status				< 0.001
Occupied	916 (51.3)	74 (77.1)	842 (49.9)	
Student	197 (11.0)	8 (8.3)	189 (11.2)	
Unoccupied	306 (17.1)	3 (3.1)	303 (17.9)	
Retired	366 (20.5)	11 (11.5)	355 (21.09)	
Enrolment group				< 0.001
General population	1312 (73.5)	26 (27.1)	1286 (76.1)	
Healthcare workers	473 (26.5)	70 (72.9)	403 (23.9)	
Smoking history				0.02
Current smoker	543 (30.4)	19 (19.8)	524 (31.0)	
Former and never smoker	1242 (69.6)	77 (80.2)	1165 (69.0)	
Smoking time (in years)^b^	20.4 ± 14.5	19.7 ± 13.7	20.5 ± 14.6	0.83
Comorbidities^c^				
At least one	1000 (56.1)	53 (55.2)	947 (56.1)	0.86
Heart disease	152 (15.2)	9 (17.0)	143 (15.1)	0.71
Vascular pathologies	64 (6.5)	4 (7.5)	60 (6.3)	0.45
Cerebrovascular disease	5 (0.5)	0 (0.0)	5 (0.5)	0.76
Diabetes mellitus	92 (9.2)	3 (5.7)	89 (9.4)	0.26
Hypertension	405 (40.5)	21 (39.6)	384 (40.5)	0.89
Respiratory pathologies	20 (2.0)	1 (1.9)	19 (2.0)	0.71
Bronchial asthma	25 (2.5)	0 (0.0)	25 (2.6)	0.25
Chronic kidney disease	8 (0.8)	0 (0.0)	8 (0.8)	0.65
Cancer	20 (2.0)	1 (1.9)	19 (2.0)	0.91
Autoimmune disease	49 (4.9)	7 (13.2)	42 (4.4)	0.004
Mental disorder/disorder	5 (5.0)	0 (0.0)	5 (0.5)	0.76
Organ transplant history	2 (2.0)	0 (0.0)	2 (0.2)	0.90
Others	681 (68.1)	39 (73.6)	642 (67.8)	0.24
Subjects with previous diagnosis of SARS-CoV-2 infection	81 (4.5)	56 (58.3)	25 (1.5)	< 0.001
Subjects hospitalized due to COVID-19	8 (0.4)	7 (7.3)	1 (0.1)	< 0.001
COVID-19-like symptoms in the period starting from 1 March 2020				< 0.001 for all categories
At least one	256 (14.3)	63 (65.6)	193 (11.4)	
Fever or a history of fever/chills	102 (5.7)	43 (44.8)	53 (3.5)	
Cough	128 (7.2)	39 (40.6)	89 (5.3)	
Shortness of breath or difficulty in breathing	88 (4.9)	43 (43.8)	46 (2.7)	
Tiredness (feeling tired without energy)	137 (7.7)	51 (53.1)	86 (5.1)	
Muscle/joint or body pains	124 (6.9)	47 (49.0)	77 (4.6)	
Ageusia (loss of sense of taste)	75 (4.2)	46 (47.9)	29 (1.7)	
Anosmia (loss of smell)	71 (4.0)	42 (43.7)	29 (1.7)	
Burning throat	89 (5.0)	22 (22.9)	67 (4.0)	
Nasal congestion or runny nose	93 (5.2)	20 (20.8)	73 (4.3)	
Diarrhoea	74 (4.1)	36 (37.5)	38 (2.2)	
Seeking medical care due to symptoms	140 (54.7)	52 (82.5)	88 (45.6)	< 0.001

*SARS-CoV-2* severe acute respiratory syndrome coronavirus 2, *IgG* immunoglobulin G, *COVID-19* coronavirus disease 2019

^a^Row percentage

^b^Summarized by mean and standard deviation (SD)

^c^Percentage was calculated on subjects with at least one chronic condition

Almost one-third the participants smoked (30.4%), while 1242 (69.6%) were classified as former or never smokers, based on the serum cotinine levels. Concordance between self-reported smoking history and serum cotinine threshold was very high, with 97.1% former smokers and 98.7% never smokers having less than 20 ng/mL cotinine level. As regards the relationship between SARS-CoV-2 infection and smoking, the prevalence of SARS-CoV-2 IgG positivity was significantly lower in current smokers (19.8%) than comparators (31.0%, *p* value = 0.02). No statistically significant association was observed between SARS-CoV-2 IgG positivity and smoking duration. Smokers had higher probability of reporting fever or chills, cough, tiredness, muscle or joint pain, burning throat, and nasal congestion (Table S1, Supplementary materials). Smokers were also more likely to seek medical care because of COVID-19-like symptoms (Table S1, Supplementary materials). Adjusting for covariates at multivariable Poisson regression, tobacco use was associated with a higher incidence rate of COVID-19-like illness, measured as the probability of having experienced at least one symptom (RR 2.45; 95% CI 1.95–3.08; *p* value < 0.001) (Table S2, Supplementary materials).

### Propensity score matching

A propensity scores for smoking status was calculated, accounting for those characteristics that were likely to have had an effect on the risk for SARS-CoV-2 infection and the imbalance of these covariates across groups. The selected characteristics included age, sex, presence of comorbidities (at least, an important chronic condition), and cohort group (as proxy of exposure risk to SARS-CoV-2). The 1:1 matching resulted in 543 matched pairs and a sample size of 1086 patients, with differences between smokers and non-smokers no longer significant in the PS-matched sample. Descriptive statistics before and after PSM, and distributions of propensity scores in smoker and comparison groups’ overlap are, respectively, in Table 3 and Fig. S1 (see Supplementary materials). Compared with before PSM, standardized group differences across all covariates were less than 0.1, representing negligible differences across age, sex, presence of comorbidities, and cohort group (Fig. S2).

**Table 3:** Baseline characteristics for smokers before and after propensity score matching

Characteristic	Total sample *N* (%)	Before matching			After matching		
		Smokers *N* (%)	Non-smokers *N* (%)	Comparison (*p* value)	Smokers *N* (%)	Non-smokers *N* (%)	Comparison (*p* value)
*N*	1785	543	1242		543	543	
Sex				0.005			0.71
Female	1096 (61.4)	307 (56.5)	789 (63.5)		307 (56.5)	313 (57.6)	
Male	689 (38.6)	236 (43.5)	453 (36.5)		236 (43.5)	230 (42.4)	
Age (continuous, in years)	50.0 ± 19.7	45.2 ± 15.0	52.0 ± 21.1	< 0.001	45.2 ± 15.0	45.2 ± 16.6	0.99
Cohort				< 0.001			0.80
General population	916 (51.3)	369 (32.0)	943 (75.9)		369 (32.0)	365 (67.2)	
Healthcare workers	197 (11.0)	174 (68.0)	299 (24.1)		174 (68.0)	178 (32.8)	
Comorbidities				< 0.001			0.54
At least one	1000 (56.1)	260 (47.9)	740 (59.6)		260 (47.9)	270 (49.7)	
None	784 (43.9)	283 (52.1)	501 (40.4)		283 (52.1)	273 (50.3)	

### Multivariate analyses after PSM

Table 4 shows the OR and 95% CI results for the logistic multivariate regression models. After PSM, Model 1 was designed to analyse the relationship between smoking and positive testing at SARS-CoV-2 serology, indicated that current smoking was associated with a decreased risk of IgG positivity (OR 0.23; 95% CI 0.12–0.045; *p* value < 0.001). An elevated risk was observed in HCWs (OR 5.45; 95% CI 2.54–11.70; *p* value < 0.001), in those with at least one chronic condition (OR 2.21; 95% CI 1.13–4.33; *p* value = 0.02), and in those who reported at least one COVID-19-like symptom (OR 13.38; 95% CI 6.72–26.64; *p* value < 0.001). As shown in Model 2 (Table 4), the adjusted odds of a diagnosis of SARS-CoV-2 infection by serology testing was significantly lower in smokers (OR 0.51; 95% CI 0.28–0.93; *p* value = 0.03), but markedly higher in HCW (OR 14.56; 95% CI 6.36–33.30; *p* value < 0.001). The final model of the multivariate exact logistic regression analysis examining the variables associated with the likelihood of hospitalization due to COVID-19 did not yield significant results (Table 4, Model 3).

**Table 4:** Logistic multivariate regression models indicating associations between positive SARS-CoV-2 serology and characteristic evaluated

Variable	Unmatched (*n* = 1784)			Matched (*n* = 1086)		
	Log likelihood = − 270.46; *χ*^2^ = 206.89 (6 *df*); *R*2: 0.28; *p* < 0.0001			Log likelihood = − 157.00; *χ*^2^ = 138.70 (6 *df*); *R*2: 0.31; *p* < 0.0001		
	OR	95% CI	*p* value	OR	95% CI	*p* value
Model 1: Likelihood of testing positive at SARS-CoV-2 serology						
Smoking status						
Never/Former smokers	Ref	–	–	Ref	–	–
Current smokers	0.23	0.13–0.41	< 0.001	0.23	0.12–0.45	< 0.001
Sex						
Male	Ref	–	–	Ref	–	–
Female	0.84	0.51–1.39	0.49	0.96	0.49–1.89	0.90
Age (continuous, in years)	0.99	0.97–1.00	0.14	0.97	0.95–1.00	0.06
Cohort						
Non-Healthcare workers	Ref	–	–	Ref	–	–
Healthcare workers	4.52	2.65–7.69	< 0.001	5.45	2.54–11.70	< 0.001
Comorbidities						
None	Ref	–	–	Ref	–	–
At least one	1.46	0.86–2.45	0.16	2.21	1.13–4.33	0.02
COVID-19-like symptoms in the period starting from March 1, 2020						
None	Ref	–	–	Ref	–	–
At least one	11.97	7.17–19.97	< 0.001	13.38	6.72–26.64	< 0.001

Variable	Unmatched (*n* = 1784)			Matched (*n* = 1086)		
	Log likelihood = − 262.20; *χ*^2^ = 134.79 (5 *df*); *R*2: 0.20; *p* < 0.0001			Log likelihood = − 171.06; *χ*^2^ = 75.41 (5 *df*); *R*2: 0.18; *p* < 0.0001		
	OR	95% CI	*p* value	OR	95% CI	*p* value
Model 2: Likelihood of previous SARS-CoV-2 infection						
Smoking status						
Never/Former smokers	Ref	–	–	Ref	–	–
Current smokers	0.50	0.29–0.89	0.02	0.51	0.28–0.93	0.03
Sex						
Male	Ref	–	–	Ref	–	–
Female	1.21	0.71–2.06	0.49	1.10	0.57–2.10	0.78
Age (continuous, in years)	1.02	1.00–1.04	0.14	1.01	0.98–1.04	0.65
Cohort						
Non-healthcare workers	Ref	–	–	Ref	–	–
Healthcare workers	18.87	10.02–35.50	< 0.001	14.56	6.36–33.30	< 0.001
Comorbidities						
None	Ref	–	–	Ref	–	–
At least one	0.95	0.57–1.61	0.86	1.16	0.62–2.18	0.65

Variable	Unmatched (*n* = 81)			Matched (*n* = 52)		
	Model Score: 4.77; *p* = 0.31			Model Score: 5.00; *p* = 0.30		
	OR	95% CI	*p* value	OR	95% CI	*p* value
Model 3: Likelihood of being hospitalized due to COVID-19						
Smoking status						
Never/Former smokers	Ref	–	–	Ref	–	–
Current smokers	1.46	0.12–11.60	0.99	1.81	0.12–25.79	0.92
Sex						
Male	Ref	–	–	Ref	–	–
Female	0.29	0.04–1.89	0.24	0.21	0.01–2.26	0.25
Age (continuous, in years)	0.98	0.90–1.06	0.60	1.07	0.92–1.29	0.47
Comorbidities						
None	Ref	–	–	Ref	–	–
At least one	3.55	0.48–43.73	0.30	4.60	0.38–277.22	0.38

*OR* odds ratio, *95% CI* 95% confidence interval, *Ref* reference category, *SARS-CoV-2* severe acute respiratory syndrome coronavirus 2, *COVID-19* coronavirus disease 2019

## Discussion

Our research is the first population-based study that used direct laboratory measures of smoking exposure, aiming at refining the association between active smoking and SARS-CoV-2 infection susceptibility. In this study, we observed a lower proportion of positive SARS-CoV-2 serology in current smokers compared with non-smokers/ex-smokers. Similarly, current smokers were less likely to have received a diagnosis of SARS-CoV-2 infection. No evidence was found about the risk of hospitalization in COVID-19 patients, likely because of the small number of hospitalized cases in our sample. We also conducted logistic regression analyses and found that the association was persistently negative even after adjusting for sex, age, previous SARS-CoV-2 infection, presence of comorbidities, and group of enrolment (as a proxy of infection risk exposure). Furthermore, the point estimates based on the PS-matched models were consistent with those for the whole study population.

Total sero-prevalence of IgG antibodies in this study was 5.4%. This proportion is in line with the results gathered in other population-based sero-epidemiological surveys conducted after the first epidemic wave in the most affected areas in Italy and elsewhere (March–June 2020), also reflecting the significant SARS-CoV-2 circulation in the Troina area [26, 47–50]. In particular, our study revealed a proportion of subjects with circulating antibodies higher than that detected by the Italian Institute of Statistics (ISTAT) during the same period, although remained below the 7.5% registered in Lombardy region in the same survey, which was hit hardest in terms of cases and death toll during between March and June 2020 [10, 26, 37]. Stratifying by cohort, the rate of sero-prevalence among HCWs peaked at 14.8%, roughly in line with similar studies that surveyed HCWs [37, 51–53]. In this regard, it should be mentioned that antibody prevalence in HCWs showed a high variability, according to different aspects of surveys design and conduction, including magnitude of SARS-CoV-2 spread in study settings, type of healthcare facilities and workers enrolled, local availability of personal protective equipment [26].

The reduced risk for confirmed SARS-CoV-2 infection in tobacco users has been previously reported. For instance, Israel described a risk reduction in current smokers [28], and a study conducted in Lombardy region confirmed the proportion of 9.2% of IgM/IgG in current smokers, compared with 19.6% of non-smokers (former and never) [26]. Compared with this previous evidence and other analogous studies [26–28], this survey allowed investigating the smoking status and history resolving problems related to self-reporting and, thus, eliminating possible bias. Furthermore, the use of community-based data also avoided selection bias associated with the use of case series, which raised questions about the representativeness of cases compared with the general population. It is worth also noting that previous research relayed on the assessment of smoking in hospitalized subjects, with a major limitation due to the lack of appropriate controls [20]. Another similar weakness can be inferred from studies that enrolled patients with confirmed SARS-CoV-2 infection, leaving outside asymptomatic or *pauci*-symptomatic individuals. Indeed many studies included online surveys, which found that the burden of COVID-19-like symptoms and self-reported SARS-CoV-2 infection were significantly associated with smoking in syndromic surveillance data [24, 34, 46, 47, 54]—consistently with our findings—with a limitation on objective identifying and quantifying of attributable symptoms [55]. In confirmation of this, Clift also highlighted that heavy smoking (i.e., above 20 cigarettes per day) was associated with a reduced risk of SARS-CoV-2 infection when weighting by the probability of having received a SARS-CoV-2 test, likely prescribed for the occurrence of overlapping symptoms [24, 46].

In contrast, current smoking has been identified as a possible risk factor for progression of the disease, and was associated with higher risks of severe COVID-19 outcomes and death in large population-based researches [24, 25].

More in general, as previously discussed, the research about the effects of active smoking on both infection and disease is still controversial [19, 21], with some methodological limitations and pitfalls found in the literature so far available, including the assessment of smoking status and history among study populations, and systematic ascertainment biases and confounders in case-series on hospitalized smokers which might have led to inaccurate determining the overall smoking-attributable risk across the research [20, 33]. Additionally, some research on the response to COVID-19 vaccines highlighted a link between smoking and the humoral response to COVID-19 vaccines with effects on IgG titre and kinetics, with smoking accelerating the decline in vaccine-induced antibodies titre [56–58]. If a similar smoking-attributable effect occurs with antibodies induced by natural SARS-CoV-2 infection, then a much lower prevalence of IgG positivity is to be expected in smokers. Taken together, these findings call for further research about the effect of smoking on COVID-19 and immunological response to both infection and vaccines [56–58].

The mechanisms by which tobacco use decreases the risk of SARS-CoV-2 infection (and increases the risk of severe prognosis in COVID-19 patients) are not fully understood. Anti-inflammatory properties mediated by α7 nicotinic acetylcholine receptors and reduction in membrane angiotensin-converting enzyme 2 (ACE-2) expression in bronchial cells—which could play a role in SARS-CoV-2 pathology—have been proposed [20, 26, 28, 59–61]. Coronaviruses bind the ACE-2 host cell receptors through homotrimeric spike protein (i.e., S1 and S2 subunit) of their envelope, and, therefore, ACE-2 expression on bronchial tissue is a strong determinant for coronaviruses infectivity. Some studies captured a significant decrease of membrane ACE-2 protein expression attributable to cigarette smoking [61–65]. Again, this effect on ACE-2 might be likely attributable to acute smoking exposure, and thus unlikely to be associated with smoking duration, as revealed by our analyses.

Furthermore, Farsalinos et al. speculated that an anti-inflammatory pathway induced by nicotinic acetylcholine receptor might modulate the immune response from hyper-inflammation stimulated in severe COVID-19 [20, 66]. The cholinergic anti-inflammatory pathway, mediated mainly through the vagus nerve, represents a reflex mechanism based on a bi-directional communication between the immune and nervous systems [67, 68].

It can restore immune homeostasis and prevent cytokine storm, a hallmark of severe COVID-19. This hypothesis warrants further study, but the authors also suggested that the cessation of nicotine intake in hospitalized smokers leads to dysregulation of the cholinergic anti-inflammatory pathway and uncontrolled immune response, and was thus responsible for higher risk for severe outcomes [20]. Recently, a pharmaceutical company reported that α7 cholinergic agonists exhibit antiviral properties both in vitro and in vivo in experimental animals (macaques), but more clinical evidence is needed to verify or reject this hypothesis [69]. Smoking is a leading cause of morbidity and mortality worldwide, and smokers should be encouraged to quit for reducing the heavy burden associated with tobacco use [15, 58]. Obviously, even if results of a low infection rate among smokers will be confirmed in further study, smoking must not be perceived as a protective measure for COVID-19, neither encouraged nor recommended. However, the possibility for therapeutic effects of nicotine or nicotinic-cholinergic agonists on COVID-19 warrants further investigation by the research community through experimental in vitro studies and in clinical trials [58, 61, 70].

This paper has a number of strengths and weaknesses. The study is the first one to use objective measure of the smoking status, thus avoiding reporting bias and allowing to precisely detecting active smoking among participants. It also uses a specific and sensitive antibody assay, which accurately correlate with SARS-CoV-2 infection. Moreover, the field collection of the samples was conducted well before the launch of the national mass vaccination campaign, an important confounder in sero-epidemiologic studies. This is a unique aspect and constitutes a non-replicable added value of this research, as future studies will not be able to discount the possible confounding role of vaccine-induced IgG.

Lastly, the cohort was carefully assembled and sample size satisfactory, being representative of the population aimed to study and thus providing reliable estimates of the association between SARS-CoV-2 infection risk and smoking.

Despite these strengths, some limitations should be acknowledged. First, the limited number of hospitalized subjects did not allow inferring conclusions on this sub-group, leaving outside important aspects related to association of smoking and COVID-19 outcomes. Second, possible recall and notoriety biases should be acknowledged regarding the self-reported COVID-19 related symptoms, for this reason we excluded some possible confounders that could have affected the reliability of the data (e.g., duration of symptoms, etc.) [46, 55]. Third, our analysis was designed to investigate sero-positivity and no relationship between IgG titres and COVID-19 outcomes or smoking could be inferred.

In conclusion, this study documents a lower proportion of positive SARS-CoV-2 serology among current smokers, using direct laboratory measures of tobacco exposure and thus avoiding possible bias associated with self-reported smoking status. As such, the research captures actionable metric on the role of smoking in SARS-CoV-2 infection and COVID-19 outcomes, and contributes to refine current epidemiological risk estimates. Results may also serve as a reference for future clinical research on potential pharmaceutical role of nicotine or nicotinic-cholinergic agonists in COVID-19.

## Supplementary Information

Below is the link to the electronic supplementary material.

Supplementary file1 (DOCX 247 kb)

## Data Availability

The data sets used and/or analysed during the current study are available from the corresponding author on reasonable request.

## Abbreviations

ACE-2: Angiotensin-converting enzyme 2
COVID-19: Coronavirus disease 2019
ELISA: Enzyme-linked immunosorbent assay
HCW: Healthcare workers
IgG: Immunoglobulin class G
IRCCS: Research Health Institute
ISTAT: Italian National Institute of Statistics
PSM: Propensity score matching
SARS-CoV-2: Severe acute respiratory syndrome—coronavirus 2
