# Supplementary material for: The effect of laboratory-verified smoking on SARS-CoV-2 infection: results from the Troina sero-epidemiological survey
Source: Intern Emerg Med. 2022 Apr 14;17(6):1617–30. doi: 10.1007/s11739-022-02975-1 (PMC9007731; doi:10.1007/s11739-022-02975-1)
Supplement: Supplementary file 1 — Supplementary file1 (DOCX 247 kb) [file 11739_2022_2975_MOESM1_ESM.docx]

## The effect of laboratory-verified smoking on SARS-CoV-2 infection: Results from the Troina sero-epidemiological survey

Supplementary Material

# Supplementary information about the propensity score matching

# *1.1 Study participants*

The Troina study enrolled two groups of participants: i) a population-based, age-stratified cohort randomly selected from the town residents; ii) a convenience group consisting of about 600 hospital staff members of Troina’s main health care establishment, where HCWs came in close contact with COVID-19 patients and therefore were exposed to higher risk of infection. These two cohorts were characterized by different baseline features (including age, sex, comorbidities), which might have influenced smoking history and habits of the enrollees, as well as posed them at a dissimilar level of risk exposure for SARS-CoV-2 infection. Therefore, in order to minimize the misclassification of risk exposure between active smokers and controls (non-smokers, namely former and never smokers), a propensity score matching was performed.

*1.2 The advantage of using propensity score matching method*

Due to the design of this study, the non-random sampling of the healthcare worker cohort and the differences among cohorts can produce selection bias and imbalance in potential confounders among different groups and their exposure to SARS-CoV-2 infection.

Logistic regression models are specified to adjust statistical association between specific variables, while the imbalance of some important confounders could be major issues in certain observational studies like the present. In fact, the sample size and the numerosity of such characteristics, collinearity, and number of covariates could influence analysis efficiency.

A propensity score is the probability that a subject would be assigned to a specific group conditional on a set of confounders, commonly predicted through logistic regression. The propensity score technique has turned out to be an effective tool to reduce or eliminate the effect of confounding factors in observational studies. Matching cases or exposed subjects against controls with similar values of propensity scores create an approximate balance for studied confounders, and the difference in outcomes between groups gives an unbiased estimate of the exposure effect.

*1.3 The calculation of the propensity score*

Considering the imbalance of the covariates across smokers’ groups, a propensity score matching (PSM) was performed, accounting for those characteristics that are likely to have an impact on the risk for SARS-CoV-2 infection. The following variables were selected: age, sex, presence of comorbidities (as at least an important chronic condition), cohort group (as proxy of exposure risk to SARS-CoV-2). The propensity scores for smoking were calculated through logistic regression models. According to optimal PSM match ratio and calliper widths for the estimation of differences in mean and proportions in observational studies, we matched the respondents on a 1:1 ratio, using the nearest neighbouring method with a calliper matching of 0.2. The analysis was carried on with R statistical software v. 3.6.2 (R Project for Statistical Computing, Vienna, Austria) and the *MatchIt* package.

The 1:1 matching resulted in 543 matched pairs and a sample size of 1086 patients: baseline characteristics before and after PSM, and distributions of propensity scores in smoker and comparison groups overlap are, respectively, in Table 3 (main text) and Fig. S1. Compared with before PSM, the absolute standardized difference across all covariates between the two groups after PSM decreased significantly. The absolute standardized difference of all covariates was ≤ 0.1 (Fig. S2).


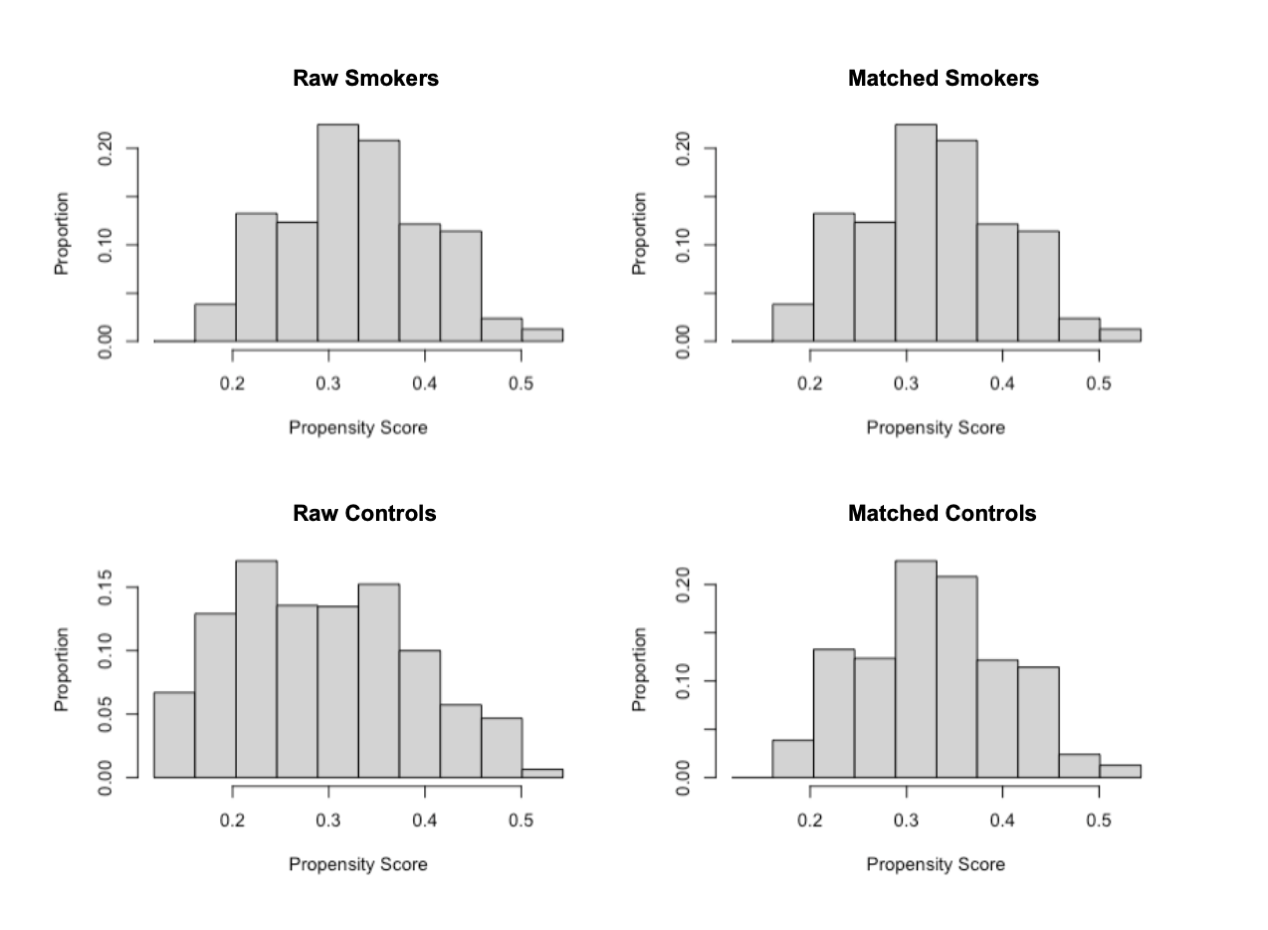


**Figure S1**. Distributions of propensity scores in smokers and comparison (non-smokers) groups overlap


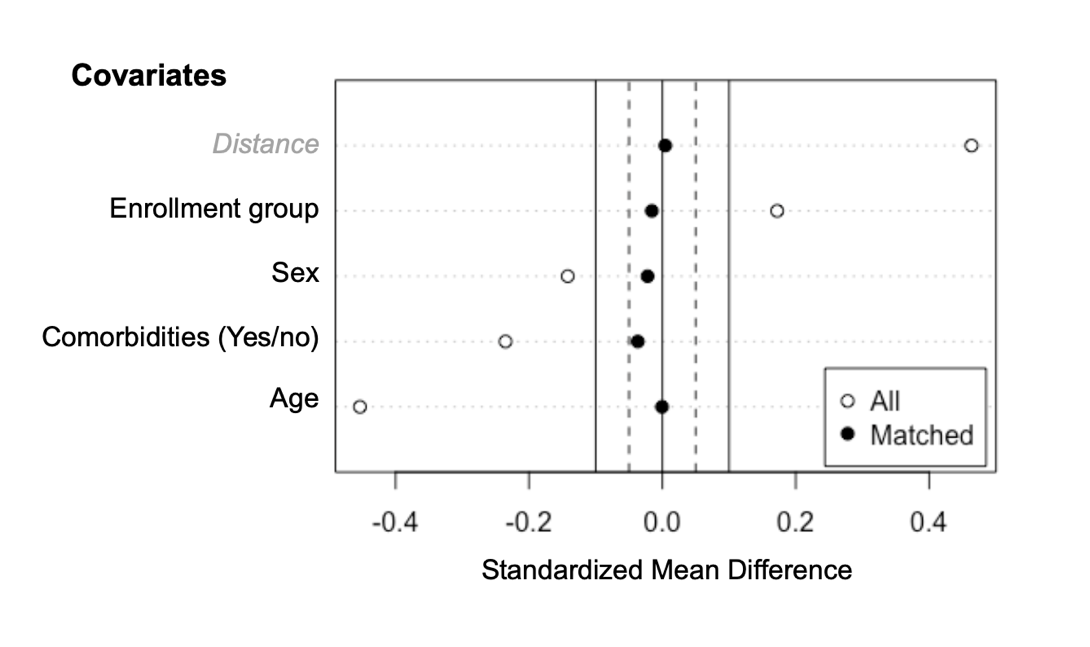


**Figure S2**. Standardized mean difference before and after propensity score matching comparing covariate values

**1.4 References** (alphabetical order)

Austin PC. An Introduction to Propensity Score Methods for Reducing the Effects of Confounding in Observational Studies. Multivariate Behav Res. 2011;46(3):399-42.

Austin PC. Optimal caliper widths for propensity-score matching when estimating differences in means and differences in proportions in observational studies. Pharm Sta. 2011;10(2):150-61.

Ho D, Imai K, King G, et all. Package ‘MatchIt’ – V. 4.3.0 (September 13, 2021). Available at: https://cran.r-project.org/web/packages/MatchIt/MatchIt.pdf.

Polosa R, Tomaselli V, Ferrara P, et al. Seroepidemiological Survey on the Impact of Smoking on SARS-CoV-2 Infection and COVID-19 Outcomes: Protocol for the Troina Study. JMIR Research Protocols. 2021;10(11):e32285. doi: 10.2196/32285.

Randolph JJ, Falbe K, Manuel AK, Balloun JL. A Step-by- Step Guide to Propensity Score Matching in R. Practical Assessment, Research & Evaluation 2014;19(18):1-6. Available at: http://pareonline.net/getvn.asp?v=19&n=18.

Rassen JA, Shelat AA, Myers J, et al. One-to-many propensity score matching in cohort studies Pharmacoepidemiol Drug Saf.. 2012;(21):69-80.

# Supplementary Tables

**Table S1**. COVID19-like symptoms distribution according to participants’ smoking status.

| **Symptom** | **Total**  **N (%)** | **Smokers**  **N (%)** | **Non-smokers**  **N (%)** | **Comparison**  **(*p-*value)** |
| --- | --- | --- | --- | --- |
| *N* | 1785 | 543 | 1242 |  |
| *At least one*  Fever or a history of fever / chills  Cough  Shortness of breath or difficulty in breathing  Tiredness (feeling tired without energy)  Muscle / joint or body pains  Ageusia (loss of sense of taste)  Anosmia (loss of smell)  Burning throat  Nasal congestion or runny nose  Diarrhea | 256 (14.3)  102 (5.7)  128 (7.2)  88 (4.9)  137 (7.7)  124 (6.9)  75 (4.2)  71 (4.0)  89 (5.0)  93 (5.2)  74 (4.1) | 131 (24.1)  44 (8.1)  63 (11.6)  31 (5.7)  58 (10.7)  53 (9.8)  27 (5.0)  26 (4.8)  43 (7.9)  43 (7.9)  24 (4.4) | 125 (10.1)  58 (4.7)  65 (5.2)  57 (4.6)  79 (6.4)  71 (5.7)  48 (3.9)  45 (3.6)  46 (3.7)  50 (4.0)  50 (4.0) | < .001  .004  < .001  .32  .002  .002  .28  .25  < .001  .001  .70 |
| Seeking any medical contact due to symptoms | 140 (54.7) | 54 (9.9) | 86 (6.9) | .03 |

**Table S2**. Association of smokers’ characteristics with the incidence of self-reported COVID-19-like symptoms in the study population.

| ***Variable*** | ***RR*** | ***SE*** | ***95% CI*** | ***p-value*** |
| --- | --- | --- | --- | --- |
| Smoking status |  |  |  |  |
| Non-smokers | *Ref.* | - | - | - |
| Current smokers | 2.45 | 0.28 | 1.95-3.08 | < .001 |
| Age (continuous, in years) | 1.00 | 0.00 | 0.99-1.01 | .84 |
| Sex |  |  |  |  |
| Male | *Ref.* | - | - | - |
| Female | 1.70 | 0.22 | 1.31-2.20 | < .001 |
| Comorbidities |  |  |  |  |
| None | *Ref.* | - | - | - |
| At least one | 0.86 | 0.12 | 0.65-1-14 | .28 |

The analysis was conducted using a multivariate Poisson regression model with log-link.

Abbreviations: *RR*, relative risk; *95% CI*, 95% confidence interval; *SE*, standard error; *Ref*, reference category.
